# Antibiotic Resistance-Susceptibility Profiles of *Streptococcus thermophilus* Isolated from Raw Milk and Genome Analysis of the Genetic Basis of Acquired Resistances

**DOI:** 10.3389/fmicb.2017.02608

**Published:** 2017-12-22

**Authors:** Ana B. Flórez, Baltasar Mayo

**Affiliations:** Departamento de Microbiología y Bioquímica, Instituto de Productos Lácteos de Asturias (CSIC), Paseo Río Linares s/n, Asturias, Spain

**Keywords:** *Streptococcus thermophilus*, lactic acid bacteria, antibiotic resistance, *ermB*, *tet*(S), whole genome sequencing, starters, horizontal gene transfer

## Abstract

The food chain is thought to play an important role in the transmission of antibiotic resistances from commensal and beneficial bacteria to pathogens. *Streptococcus thermophilus* is a lactic acid bacterium of major importance as a starter for the dairy industry. This study reports the minimum inhibitory concentration (MIC) of 16 representative antimicrobial agents to 41 isolates of *S. thermophilus* derived from raw milk. Strains showing resistance to tetracycline (seven), erythromycin and clindamycin (two), and streptomycin and neomycin (one) were found. PCR amplification identified *tet*(S) in all the tetracycline-resistant strains, and *ermB* in the two erythromycin/clindamycin-resistant strains. Hybridisation experiments suggested each resistance gene to be located in the chromosome with a similar genetic organization. Five antibiotic-resistant strains -two resistant to tetracycline (St-2 and St-9), two resistant to erythromycin/clindamycin (St-5 and St-6), and one resistant to streptomycin/neomycin (St-10)- were subjected to genome sequencing and analysis. The *tet*(S) gene was identified in small contigs of 3.2 and 3.7 kbp in St-2 and St-9, respectively, flanked by truncated copies of insertion sequence (IS) elements. Similarly, *ermB* in St-6 and St-5 was found in contigs of 1.6 and 28.1 kbp, respectively. Sequence analysis and comparison of the largest contig showed it to contain three segments (21.9, 3.7, and 1.4 kbp long) highly homologous to non-collinear sequences of pRE25 from *Enterococcus faecalis*. These segments contained the *ermB* gene, a transference module with an origin of transfer (*oriT*) plus 15 open reading frames encoding proteins involved in conjugation, and modules for plasmid replication and segregation. Homologous stretches were separated by short, IS-related sequences, resembling the genetic organization of the integrative and conjugative elements (ICEs) found in *Streptococcus* species. No gene known to provide aminoglycoside resistance was seen in St-10. Four strain-specific amino acid substitutions in the RsmG methyltransferase were scored in this strain; these might be associated to its streptomycin/neomycin resistance. Under yogurt manufacturing and storage conditions, no transfer of either *tet*(S) or *ermB* from *S. thermophilus* to *L. delbrueckii* was detected. The present results contribute toward characterisation of the antibiotic resistance profiles in *S. thermophilus*, provide evidence for the genetic basis of acquired resistances and deepen on their transference capability.

## Introduction

Resistance to antimicrobials needed in human and veterinary medicine (mostly antibiotics) is a global problem (Watkins and Bonomo, 2016). The finding of antibiotic-resistant bacteria in different foods (Wang et al., 2012) has led to concerns regarding the impact of food bacteria as reservoirs of resistance genes, and how they may be involved in the spread of antibiotic resistance genes (Marshall and Levy, 2011). In recent years, different authors have characterized antibiotic resistance determinants in dairy-associated bacteria (Ammor et al., 2007; Devirgiliis et al., 2013; Soares-Santos et al., 2015), and directly in total DNA from dairy products (Devirgiliis et al., 2008; Flórez et al., 2014). Antibiotic resistance genes tend to settle on mobile genetic elements with great transfer capacity (Brown-Jaque et al., 2015). Not surprisingly, resistance determinants identified in environmental bacteria have been recognized as identical to those found in pathogens (Davies and Davies, 2010; Forsberg et al., 2012). The complex microbial interactions that take place during the manufacture and ripening of fermented dairy products (Irlinger and Mounier, 2009), and the contact of dairy microorganisms after ingestion with the dense microbial populations of the gut (Qin et al., 2012), provide ideal scenarios in which horizontal gene transfer events can occur.

*Streptococcus thermophilus* is a lactic acid bacterium (LAB) dominant throughout the manufacture and ripening of traditional dairy products such as yogurt, yogurt-like fermented milks, and some Italian and Swiss cheese types (Parmesan, Grana, Emmental, Gruyère, etc.). The production of these products involves incubation at high temperature (>40°C) or a heating step which selects for thermophilic organisms (Parente and Cogan, 2004). In dairy fermentations, *S. thermophilus* rapidly converts lactose into lactic acid, which imparts a fresh acidic flavor and helps to suppress growth of acid-susceptible pathogens and spoilage microorganisms (Cui et al., 2016). Like other LAB species, *S. thermophilus* metabolizes milk proteins, giving rise to key flavor compounds. This property has broadened the use of this organism as an adjunct culture for cheeses manufactured using mesophilic starters (such as Cheddar, Gouda, Manchego, etc.) (Cui et al., 2016). Not surprisingly, *S. thermophilus* is considered the second-most economically important LAB species after *Lactococcus lactis* (Mills et al., 2010). The European Food Safety Authority (EFSA) includes *S. thermophilus* in its list of microorganisms with Qualified Presumption of Safety (QPS) status (EFSA, 2017). The qualification of *S. thermophilus* strains as starters therefore only requires the absence of transmissible antimicrobial resistances be confirmed (Delorme, 2008; EFSA, 2017). However, a number of *S. thermophilus* strains resistant to antibiotics have already been detected and their resistance determinants characterized (Tosi et al., 2007; Rizzotti et al., 2009; Arioli et al., 2014). In the search for new starter candidates, strains showing resistances are excluded from selection pipelines (Delorme, 2008). The characterisation of the genetic basis of antimicrobial resistance may provide clues regarding how resistance-spreading mechanisms operate, and therefore help in the design of strategies aimed at preventing antibiotic resistance gene transfer (Rossi et al., 2014).

The aims of the present work were: (i) to determine the antibiotic resistance/susceptibility profiles of a set of *S. thermophilus* isolates derived from raw milk; (ii) to investigate the genetic basis of the resistances encountered by conventional PCR and whole genome sequencing analysis; and (iii) to examine the capacity of selected antibiotic resistance genes to transfer horizontally from *S. thermophilus* to *Lactobacillus delbrueckii* subsp. *bulgaricus* during the manufacture and storage of yogurt.

## Materials and methods

### Bacterial strains and growth conditions

Forty one wild isolates of *S. thermophilus* were selected from among a collection of 106 previously obtained from raw milk samples by a selective procedure (Delgado et al., 2013). *Lactobacillus plantarum* ATCC 14917^T^ (= LMG 6907^T^) and *Enterococcus faecalis* ATCC 29212 (= LMG 8222) were used as quality control strains for antibiotic susceptibility analysis. *Lactobacillus delbrueckii* subsp. *bulgaricus* LMG 6901 and CECT 4005 were used as resistance gene recipients during experimental yogurt manufacture. *L. plantarum* LMG 6907^T^, *E. faecalis* LMG 8222, *L. delbrueckii* subsp. *bulgaricus* LMG 6901, and *S. thermophilus* LMG 9689^T^ were obtained from the Belgian Coordinate Collections of Microorganisms (LMG Bacteria Collection) (BCCM, University of Ghent, Belgium). *L. delbrueckii* subsp. *bulgaricus* CECT 4005 was obtained from the Spanish Collection of Microorganisms (CECT, University of Valencia, Spain). Unless otherwise stated, *S. thermophilus* strains were grown statically under anaerobic conditions in M17 (Oxoid, Basingstoke, UK) with 0.5% lactose (LM17) or Mueller-Hinton (Oxoid) at 37°C for 24–48 h. *L. plantarum* and *E. faecalis* were grown under aerobic conditions at 30°C in de Man Rogosa and Sharpe (MRS) broth (Merck, Darmstad, Germany). *Lactobacillus delbrueckii* subsp. *bulgaricus* strains were grown under anaerobic conditions in MRS at 37°C without shaking.

### Minimum inhibitory concentration of antibiotics

The MICs of 16 representative antibiotics were determined according to ISO Standard 10932:2010 (IDF, 2010) using VetMIC plates for LAB (National Veterinary Institute, Uppsala, Sweden). Briefly, individual colonies grown on Mueller-Hinton agar plates were suspended in 2 mL sterile saline solution (Oxoid) to obtain a density corresponding to McFarland standard 1 (≈3 × 10^8^ cfu/mL). This suspension was further diluted 1:1,000 in Mueller-Hinton broth to a final concentration of about 3 × 10^5^ cfu/mL. One hundred microliter of this inoculum were then added to each well of the VetMIC plate. The VetMIC plates contained serial 2-fold dilutions of the antibiotics ampicillin, ciprofloxacin, clindamycin, chloramphenicol, erythromycin, gentamicin, kanamycin, linezolid, neomycin, penicillin, rifampicin, streptomycin, tetracycline, trimethoprim, vancomycin, and virginiamycin. MICs were visually read after 48 h of incubation at 37°C. For some antibiotics the concentration range of the VetMIC plates proved insufficient; their MICs were determined using the MICE system (Oxoid), following the manufacturer's recommendations.

An isolate was considered phenotypically resistant to an antibiotic when it was not inhibited at a concentration equal to or higher than the established ecological cut-off (ECOFF) as defined by EFSA (EFSA, 2012).

### DNA extraction and search for antibiotic resistance genes by PCR

Total genomic DNA was extracted from *S. thermophilus* isolates using the GenElute Genomic Bacterial DNA Purification kit (Sigma-Aldrich, St. Louis, Mo., USA), following the manufacturer's instructions. The presence of tetracycline resistance genes in resistant isolates was investigated by PCR using the universal primers for genes encoding ribosomal protection proteins DI-DII (Clermont et al., 1997) and Tet1-Tet2 (Barbosa et al., 1999), and specific primers for *tet*(W) (Scott et al., 2000) *tet*(M), *tet*(S), *tet*(O), *tet*(K), and *tet*(L) (Gevers et al., 2003). The PCR conditions were those described in the cited literature. Genes associated with erythromycin resistance (*ermA, ermB, ermC, msrA, ermF*, and *mefA*) were sought by PCR using the primers and conditions as reported by Hummel et al. (2007) and Rizzotti et al. (2009). Amplicons were then subjected to electrophoresis, visualized by staining with ethidium bromide (0.5 μg mL^−1^), and photographed. Selected amplicons were purified using the GenElute PCR Clean-up kit (Sigma-Aldrich), sequenced in an ABI 373 DNA sequencer (Applied Biosystems, Waltham, Ma., USA), and the sequences obtained compared to those in the NCBI database using Blast software (https://blast.ncbi.nlm.nih.gov/Blast.cgi).

### PCR typing of isolates

Isolates were typed according to their RAPD and rep-PCR fingerprinting profiles using primer M13 (5′-GAGGGTGGCGGTTCT-3′) as reported by Rossetti and Giraffa (2005), primer BoxA2R (5′-ACGTGGTTTGAAGAGATTTTCG-3′) as reported by Koeuth et al. (1995), and primer OPA18 (5′-AGGTGACCGT-3′) as reported by Mättö et al. (2004). RAPD and rep-PCR amplifications were independently performed in 25 μL volumes containing 12.5 μL MasterMix (Ampliqon, Odense, Denmark), 5 μL of either primer (10 μM), 3 μL of purified DNA, and molecular grade water (Sigma-Aldrich). The DNA amplification involved one cycle of 95°C for 7 min, followed by 40 cycles of denaturation (at 90°C for 30 s), primer annealing (at 42°C [M13], 40°C [BoxA2R] or 32°C [OPA18] for 1 min), and extension at 72°C for 4 min. A final extension step at 72°C for 10 min was then performed. Typing reaction products were subjected to electrophoresis and recorded as above. GeneTools software v.4.03 (SynGene, Cambridge, UK) was used to compare the profiles. Patterns were clustered using the unweighted pair group with arithmetic mean (UPGMA) method, and pattern similarity expressed via the simple matching (SM) coefficient.

### DNA hybridisation

Total DNA from erythromycin- and tetracycline-resistant strains was digested with either HindIII or PstI restriction enzymes (EURx, Gdansk, Poland). After electrophoresis, the DNA was blotted onto Hybond-N nylon membranes (GE Healthcare, Buckinghamshire, UK) using a standard protocol (Sambrook and Russell, 2001). Internal segments of the *ermB* and *tet*(S) genes, both amplified by PCR, were independently used as probes after labeling with digoxigenin using the non-radioactive DIG-High Prime DNA Labeling and Detection Starter kit II (Roche, Basel, Switzerland). Labeling, hybridisation under high-stringency conditions, and detection were performed following the manufacturer's recommendations. Hybridisation signals were detected by chemoluminescence using an ImageQuant 350 Digital Imaging System (GE Healthcare, Pittsburgh, PA, USA).

### Genome sequencing and annotation

A genomic library of 0.5 kbp was constructed from total DNA belonging to five antibiotic resistant *S. thermophilus* strains, and paired-end sequenced using a HiSeq 1000 System sequencer at the Beijing Genomics Institute (BGI, Shenzhen, China). Quality-filtered reads were assembled in contigs using Spades software v.3.6.2. (http://cab.spbu.ru/software/spades/) (Bankevich et al., 2012). Genomes were annotated using the RAST annotation system (http://rast.nmpdr.org/) and the NCBI Prokaryotic Genome Annotation Pipeline (http://www.ncbi.nlm.nih.gov/genome/annotation_prok/). DNA and deduced protein sequences of interest were examined for homology using the on-line Blast program as above. The homology of DNA and proteins was further investigated by searching databases such as CARD (http://arpcard.mcmaster.ca/), KEGG (http://www.genome.jp/kegg/pathway.html), Uniprot (http://www.uniprot.org) and COG (http://www.ncbi.nlm.nih.gov/COG).

### Core genome, pangenome, and phylogenetic analyses

A comparative genome analysis of the five sequenced *S. thermophilus* strains in this study and 32 publicly-available genome sequences at the NCBI database was performed. The pan-genome of *S. thermophilus* was inferred with the Roary software program version 3.7.0 (Page et al., 2015). For this, GenBank and RAST server files were converted to GFF3 format using the Bio::Perl script bp_genbank2gff3.pl. GFF3 files were then used as an input to Roary. Core and accessory gene numbers were inferred from Roary data and a phylogenetic tree in Newick format were constructed. For visualization, Roary outputs were passed through the interactive on-line tool iTOL (Letunic and Bork, 2011).

### Analysis of transfer during milk fermentation

Mating trials were performed to test the transferability of the *ermB* and *tet*(S) genes from *S. thermophilus* to two *L. delbrueckii* subsp. *bulgaricus* strains during the manufacture and storage of yogurt-like fermented milks. Donor and recipient were independently grown overnight in UHT milk (CAPSA, Siero, Spain). These cultures were used to inoculate fresh UHT milk (2% for each donor and recipient). Inoculated milk was incubated overnight at 42°C and the fermented products stored at 4°C for up to 15 days. Donor and recipient were counted in LM17 and MRS, respectively. *Lactobacillus* transconjugants were selected for on MRS agar (Oxoid) supplemented with appropriate antibiotics (20 μg mL^−1^ tetracycline and 5 μg mL^−1^ erythromycin as required). Experiments were repeated twice.

### Genbank accession numbers

The genome sequences of *S. thermophilus* St-2, St-5, St-6, St-9, and St-10 were deposited in the GenBank database under the BioProject PRJNA419299 with biosample accession numbers SAMN08049010 to SAMN0804914, respectively.

## Results

Table 1 shows the MICs of 16 antibiotics to 41 *S. thermophilus* strains of this study. Analysis of these MICs and comparison with data in the literature indicated that seven isolates were resistant to tetracycline (MIC ≥ 8 μg mL^−1^), two resistant to both erythromycin and clindamycin (MICs 128 and ≥16 μg mL^−1^, respectively), and one resistant to streptomycin (MIC 128 μg mL^−1^). Although a cut-off for neomycin in *S. thermophilus* has yet to be established (EFSA, 2012), the MIC of this antibiotic for the streptomycin-resistant isolate was much higher than those recorded for all other isolates (256 compared to 2–32 μg mL^−1^). This suggested this strain was resistant to both streptomycin and neomycin. Analysis of the distribution of the MICs, which follows statistical normality in the case of intrinsic resistance (Murray et al., 2003), led to the same conclusions being drawn regarding the number and types of resistant isolates -except for one isolate, for which the MIC of tetracycline was only one dilution higher than the cut-off (8 μ mL^−1^ compared to 4 μ mL^−1^) (Table 1).

**Table 1:** Distribution of Minimum Inhibitory Concentrations (MICs) as determined by broth microdilution of 16 antibiotics to 41 *Streptococcus thermophilus* strains isolated from raw milk.

**Antibiotic**	**EFSA's cut-offs (μg mL^−1^)**	**Number of strains with the following MICs (**μ**g mL**^**−1**^**)**												
		**≤0.06**	**0.12**	**0.25**	**0.5**	**1**	**2**	**4**	**8**	**16**	**32**	**64**	**128**	**256**
Gentamicin	32					10	21	8	2					
Kanamycin	64								2	12	18	9		
Streptomycin	64							3	20	13	4		1^a^	
Neomycin	–						6	18	9	4	3			**1^a^**
Tetracycline	4			3	8	17	5	1	1		**3**	**3**		
Erythromycin	2	23	10	6									**2^b^^,^^c^**	
Clindamycin	2	35	3		1					**2^c^^,^^d^**				
Chloramphenicol	4					1	29	11						
Ampicillin	2	10	17	9		3	2							
Penicillin G	–	13	15	5	4	2	1	1						
Vancomycin	4			3	34	2	1	1						
Virginiamycin	–		10	23	8									
Linezolid	–				2	34	5							
Trimethoprim	–									1	12	13	15^d^	
Ciprofloxacin	–			1		5	17	14	4					
Rifampicin	–	7	5	18	9	2								

*Gray-shaded boxes show strains considered resistant in this work*.

a *The strain with the highest MIC to both streptomycin and neomycin was the same*.

b *These two strains were able to grow at the highest erythromycin concentration assayed in the microdilution assay using the VetMIC plates (8 μg mL^−1^); the current MIC value was obtained in a subsequent E-test assay, testing a larger range of antibiotic concentrations*.

c *The two strains resistant to erythromycin and clindamycin were the same*.

d *The highest concentration assayed for clindamycin and trimethoprim in the microdilution assay was 8 and 64 μg/ml, respectively; MICs of these two antibiotics should be read as ≥16 and ≥128 μg/ml, respectively*.

**Figure 1:**
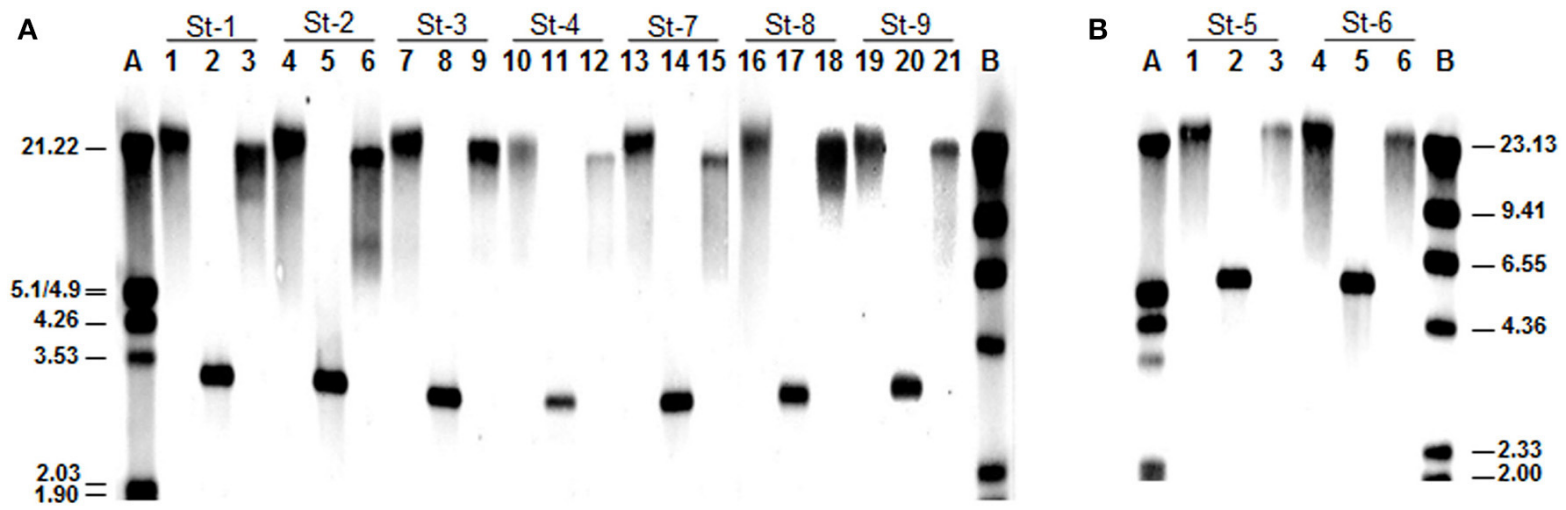
Southern blot analysis of total genomic DNA undigested and digested with either HindIII and PstI from the *S. thermophilus* strains resistant to tetracycline **(A)** and erythromycin **(B)**, respectively. As a probe, internal segments of *tet*(S) (in **A**) and *ermB* (in **B**) genes obtained by specific PCR and labeled with digoxigenin (Roche) were used. Molecular weight markers: A, digoxigenin-labeled, EcoRI and HindIII-digested lambda DNA; B, digoxigenin-labeled, HindIII-digested lambda DNA. Size of the fragments (in kbp) is indicated. The code numbers of the resistant strains are given above the lane numbers.

To detect the genetic determinant(s) responsible for resistance to tetracycline and erythromycin, the presence of well-known genes associated with resistance to these antibiotics was investigated by PCR using universal and gene-specific primers. With the universal primer pairs DI-DII or Tet1-Tet2, all isolates resistant to tetracycline returned positive amplifications. Further analysis showed them all to produce an amplicon when using primers specifically targeting the *tet*(S) gene. The sequenced amplicons were nucleotide-identical among themselves and to some database-held sequences of *tet*(S) genes from different species. Similarly, the two isolates resistant to erythromycin produced amplicons with nucleotide sequences identical to one another and to *ermB* gene sequences in databases. Since no correlation between genes and aminoglycoside resistance appears to exist in many LAB species (Ammor et al., 2008), no genes associated with this phenotype were searched for using PCR amplification. Hybridisation experiments using internal segments of *tet*(S) or *erm*B as a probe were performed to identify the genetic location of the antibiotic resistance isolates. Figure 1 shows the results obtained. The finding of hybridisation signals in undigested and digested (HindIII and PstI) total DNA at around the same positions in isolates harboring *tet*(S) (Figure 1A) or *ermB* (Figure 1B) suggests the genetic organization of the genes in each of the resistant isolates to be identical, or at least very similar. No plasmid bands were detected in any of the resistant isolates (data not shown); both *tet*(S) and *ermB* genes were therefore considered to be located in the bacterial chromosome.

The isolates resistant to tetracycline (seven) and erythromycin (two) were then subjected to molecular typing by the rapid amplification of polymorphic DNA (RAPD) technique using three different primers in order to assess whether they corresponded to different strains or to replicates (Supplementary Figures 1A–C). Given the reproducibility of the assay (>92%; data not shown), isolates sharing a percentage similarity of >90% (an arbitrary figure) were considered to be the same strain. Under these experimental conditions, six different strains were contemplated, of which four showed tetracycline resistance and two erythromycin resistance (Supplementary Figure 1D). Because of its distinctive phenotype, the isolate resistant to streptomycin was considered an independent strain.

Based on the phenotypic, hybridisation and typing results, five strains (St-2 and St-9 resistant to tetracycline, St-5 and St-6 resistant to erythromycin/clindamycin, and St-10 resistant to streptomycin/neomycin) were subjected to genome sequencing. The general data of the genome sequencing projects of the five *S. thermophilus* strains are summarized in Supplementary Table 1. In general, similar results were returned for each strain. The size of the genomes was, in all cases, around 1.9 Mbp and the number of contigs ranged between 56 and 79. The key genetic features of the genomes discovered during sequence analysis are summarized in Supplementary Table 2. Single genes (no sequence heterogeneity) coding for the three rRNA molecules (23S, 16S, and 5S) were found in all strains. Several bacteriocin-like gene clusters were identified in all strains too, as were some genes coding for proteins homologous to others considered to be toxins (exfoliative toxin, protein J, doc toxin, etc.) produced by pathogenic staphylococci and streptococci. A vast array of genes dedicated to transport and degradation of sugars and polysaccharides was also recorded. Despite the dairy origin of the isolates, no genes coding for a caseinolytic proteinase similar to PrtS (Delorme et al., 2010) were identified (Supplementary Table 2). A comparative analysis of the genome sequences of the five *S. thermophilus* strains of this study and 32 from GenBank (Supplementary Table 3) was undertaken to provide knowledge on the genotype characteristics of the strain and their evolutionary relationships. The pan-genome of all 41 strains presented a total of 5,165 genes, of which a set of 655 genes were present in >99% of the strains (core genome). As shared genes, a group of 1,690 genes were identified in 15–95% of the strains, and 2,820 genes were present in less than 15% of the strains. Focusing on the antibiotic resistant strains, the Roary analysis identified a range of unique genes (from 18 to 81) among the strains of this study (Supplementary Table 2). Based on the gene content obtained with Roary, the phylogenomic analysis of 32 representative strains of *Streptococcus thermophilus* available at the NCBI database and the five wild *S. thermophilus* antibiotic resistant strains of in this study, separated the strains in three unrelated clades (Supplementary Figure 2). The antibiotic resistance strains were allocated to clade A (one strain) and clade B (four strains). Though they all could be distinghished by some genomic features (Supplementary Table 2), tetracycline and erythromycin resistant strains proved to be close relatives, particularly St-5 and St-6 (the two erythromycin resistant strains). Clade C included a majority of the strains; these have mostly been isolated from commercial and natural starters and proved to be phylogenetically distant to the wild strains of this study.

As expected, genome analysis also confirmed the presence of *tet*(S) and *ermB* in the tetracycline- and erythromycin-resistant strains in which they were previously detected by PCR. In addition to these, 20, 14, 14, 18, and 15 genes in strains St-2, St-5, St-6, St-9, and St-10 respectively, were classified by the RAST server as belonging to the category “Virulence, Disease, and Defense,” subcategory “Resistance to Antibiotic and Toxic Compounds.” The majority of the latter genes encoded components dedicated to homeostasis and/or resistance to heavy metals such as arsenic, copper, mercury, and the cobalt-zinc-cadmium triad. Further, ORFs encoding elongation factors, efflux pumps, and DNA gyrases and topoisomerases were also included by RAST in this subcategory. Neither the gene content (presence of genes of concern [as known from databases] in the genome of most strains) nor the gene context (flanked by housekeeping-like genes in the absence of DNA sequences related to mobile genetic elements in the neighborhood) suggested any of them to be a true, dedicated antibiotic resistance gene. No gene known to be involved in aminoglycoside resistance in LAB was identified in the streptomycin/neomycin-resistant strain (St-10).

**Figure 2:**
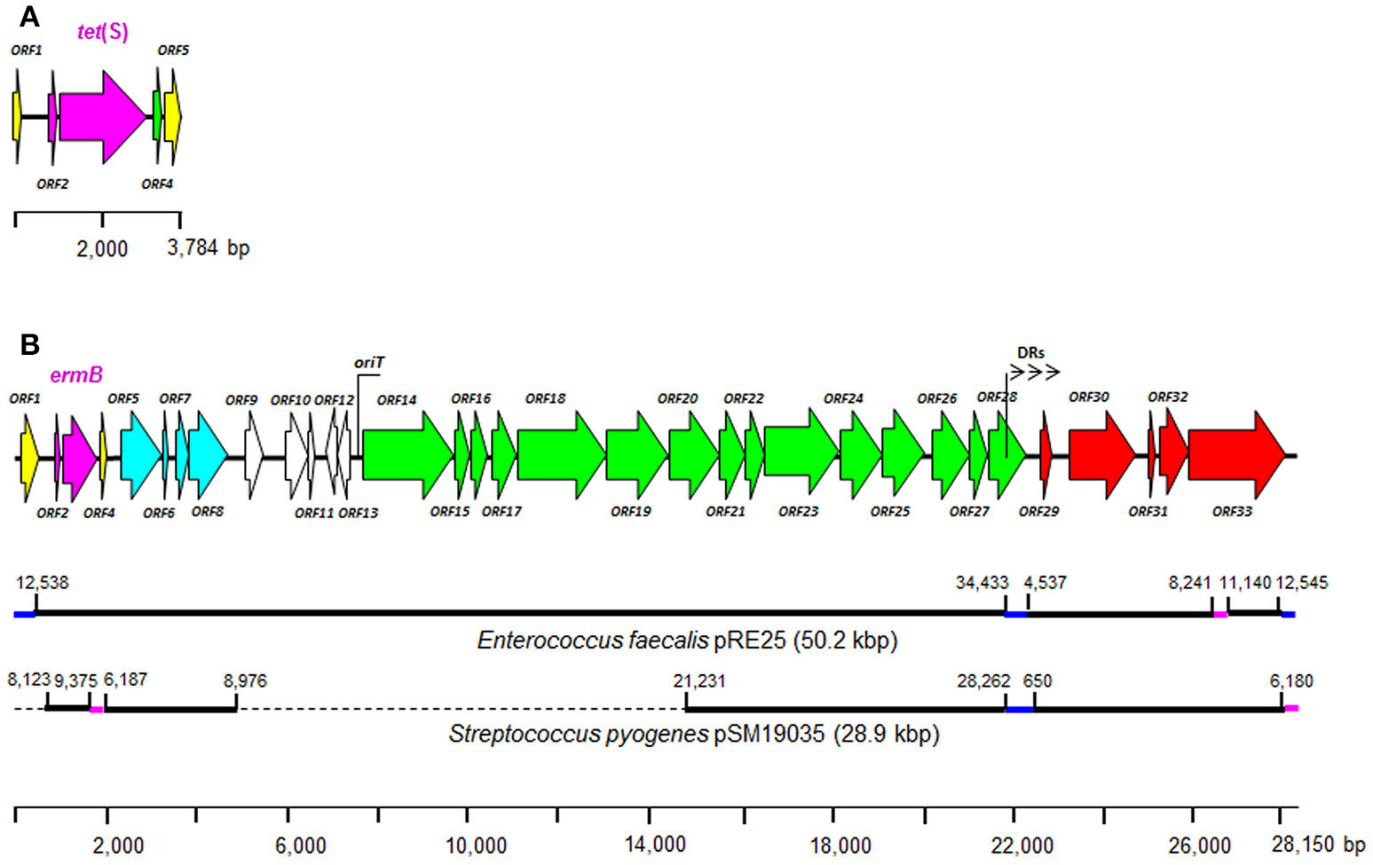
Genetic organization of ORFs in the contig harboring the tetracycline resistance gene *tet*(S) from the genome sequence of *S. thermophilus* St-9 **(A)** and in the contig harboring the erythromycin resistance gene *ermB* from the genome of *S. thermophilus* St-5 **(B)**. Color code of the ORFs: in yellow, ORFs complete and/or incomplete ORFs related to transposases, invertases, and topoisomerases; in purple, ORFs involved in antibiotic resistance; in light blue, ORFs related to plasmid segregation and stability; in green, ORF involved in conjugation/mobilization; in white, ORFs encoding hypothetical proteins or proteins coding for other systems. Specific features of **(B)**: (i) the position of *oriT* is also indicated; (ii) arrowheads represent a region of direct repeats (DR) in front of the replication module, which showed no significant homology to those in pRE25 and pSM19035; (iii) long segments of the contig (black bars) highly similar (>99% nucleotide identity) or identical to plasmid sequences of pRE25 from *Enterococcus faecalis* and pSM19035 from *Streptococcus pyogenes*, as indicated; (iv) numbering in plasmids indicates start and end positions of regions of homology to sequences in the contig; (v) purple bars, constituted by truncated insertion sequence (IS) elements, separate non-colinear regions of homology; (vi) blue bars indicate regions of low homology to those found in pRE25 and pSM19035; (vii) the broken line indicates sequences not present in pSM19035.

*tet*(S) was found in strains St-2 and St-9 in two similarly-organized short contigs of 3,224 and 3,784 bp respectively. The genetic organization of the largest contig is depicted in Figure 2A. Review of the hybridisation results gave no further clues regarding the genetic organization of these genes, as the ≈3.3 kbp HindIII fragment included a major part of *tet*(S) and extended beyond the sequence in the contigs. In both cases, the genes were shown to be flanked by fragments of insertion sequence (IS)-like elements. From position 98 onwards, the sequence of the shortest contig was identical to that in the largest but 246 bp smaller. Nevertheless, in both contigs, *tet*(S) was flanked on both sides by truncated copies of the same IS-derived elements: a fragment of an IS256-like element at the 5′ end, and an IS6-like fragment at the 3′ end. Immediately upstream of *tet*(S) an inverted repeat of 16 bp was found (Supplementary Table 4) overlapping a DNA sequence encoding a peptide with amino acid similarity to an internal segment of the *tet*(M) leader peptide (Su et al., 1992); this peptide has been shown to be involved in transcriptional attenuation.

Similar to that seen for *tet*(S), the *ermB* genes in St-5 and St-6 were identified in contigs of 28,150 and 1,615 bp respectively. Except for the last nucleotide at the 3′ end, the whole sequence of the contig from St-6 overlapped that of St-5. The segment shared by the two strains contained little more than the *ermB* and MLS leader peptide-encoding genes, and was flanked by short truncated segments of transposase- and topoisomerase-related sequences. The genetic organization of the largest contig is depicted in Figure 2B, and the analysis of open reading frames (ORFs) and other contig-specific features is summarized in Supplementary Table 4. The contig sequence showed strong nucleotide identity to large segments of plasmid sequences, particularly to three long, non-contiguous stretches of 21.9, 3.7, and 1.4 kbp long to the 50.2 kbp plasmid pRE25 from *Enterococcus faecalis* (GenBank accession no. NC_008445.1). It also showed strong homology to segments of the 28.9 kbp plasmid pSM19035 (NC_006979.1) from *Streptococcus pyogenes* (Figure 2B). The homologous segments were flanked by truncated copies of IS elements, which were repeated at several positions in both pRE25 and pSM19035. As in pRE25, four ORFs (ORF5 to ORF8 in Figure 2B) related to plasmid segregation and stability were identified downstream of the *ermB* gene, followed by a series of genes encoding hypothetical proteins. Further downstream, a long segment of DNA containing an *oriT* sequence followed by 15 ORFs (ORF14 through ORF28) was also noted. The latter segment is thought to be the minimal conjugative unit of pRE25; it is also present in pSM19035 and other conjugative plasmids such as pIP501 (Kurenbatch et al., 2002). Downstream of the conjugation module, a series of three 9 bp-long direct repeats (DRs) was identified: DR1 (TCCAGTTGA) was repeated 8 times, DR2 (CCAACAGAG) 12 times, and DR3 (CCAACGGAA) 21 times. DR1 and DR2 were separated by just one thymine (T) base, and repeats of DR2 and DR3 seemed to be interspersed at random. These DRs were found in front of five ORFs involved in plasmid replication and copy number control (Figure 2B). The nucleotide similarity within these ORFs to sequences in pRE25 and pSM19035 (96%) was more conserved than within the region of the DRs, where no significant homology was found.

**Figure 3:**
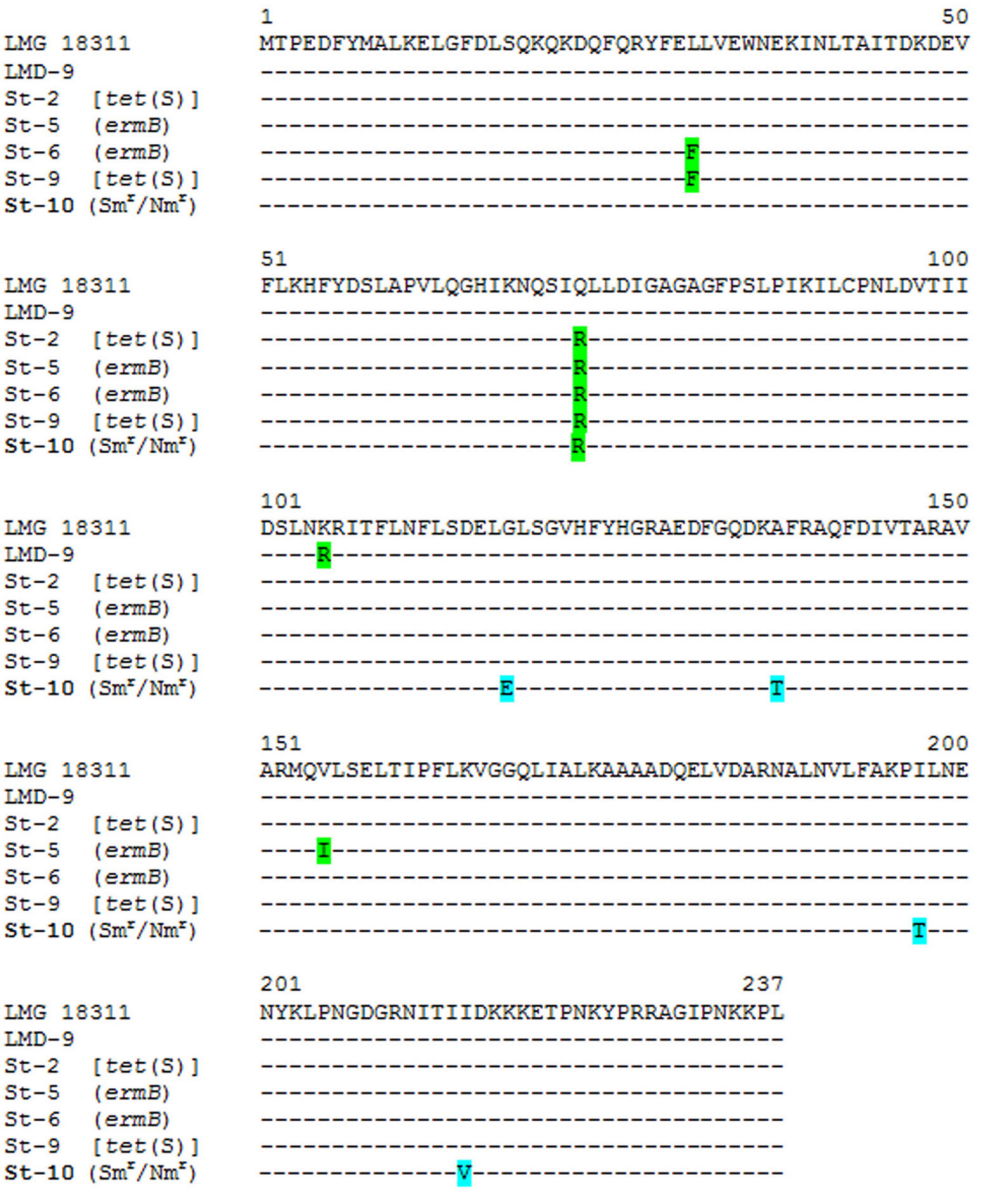
Alignment of the deduced amino acid compositions of the 16S rRNA guanine(527)-N(7)-methyltranferases encoded by *rsmG* genes from the different *S. thermophilus* strains of this study. As a reference, the deduced sequence of RsmG protein from *S. thermophilus* LMG 18311 (GCA_000011825.1) and LMD-9 (GCA_000014485.1) were used. Amino acid changes in the streptomycin resistant strain *S. thermophilus* St-4 are highlighted in pale blue. In green, other amino acid changes in streptomycin susceptible strains.

Genome analysis of St-10 revealed no genes known to provide resistance to streptomycin and/or neomycin. Therefore, genes in which mutations are known to cause resistance to aminoglycosides (such as those coding for the 16S rRNA molecule and the ribosomal protein S12) were analyzed and compared among all sequenced strains and to those in databases. No heterogeneity was detected in the gene encoding the 16S rRNA; all five sequences from the present strains were identical, and indeed identical to those of most strains in databases. A nucleotide change giving rise to an amino acid difference was observed in the S12 protein in the streptomycin/neomycin resistant strain St-10, but the same mutation was also present in the streptomycin- and neomycin-susceptible strain St-2 (one of the tetracycline-resistant strains). Further, this change is far away (T132A) from those known to be involved in aminoglycoside resistance (positions 42, 43, and 87) (Carter et al., 2000). In *Bacillus subtilis* (Nishimura et al., 2007) and *Thermus thermophilus* (Demirci et al., 2014), low level of streptomycin resistance has been attributed to deficient N-7 methylation of the 16S rRNA molecule at position G527 by the product of the *rsmG* gene. Alignment of the deduced RsmG proteins from all sequenced strains in this study and others from databases, showed heterogeneity at several positions (Figure 3). In particular, the protein sequence from the streptomycin/neomycin-resistant strain (St-10) showed four exclusive amino acid changes at positions 118 (G → E), 137 (A → T), 197 (I → T), and 215 (I → V). One or more of these amino acid changes might reduce or abolish the methylating activity of RsmG, destabilizing the conformation of the streptomycin binding sites (Demirci et al., 2014), thus increasing resistance to this antibiotic and other aminoglycosides.

The transferability of *tet*(S) and *ermB* from *S. thermophilus* to *L. delbrueckii* strains was independently conducted under yogurt manufacturing conditions. Ten different batches of yogurt were produced using as starters two *L. delbrueckii* subsp. *bulgaricus* strains in combination with three *S. thermophilus* strains carrying *tet*(S), and two *S. thermophilus* strains carrying *ermB* (Supplementary Figure 3). The transfer of antibiotic resistance after milk fermentation and during yogurt storage (for up to 15 days) was checked in MRS agar containing appropriate antibiotics. However, no *L. delbrueckii* colonies resistant to either tetracycline or erythromycin were ever recovered.

## Discussion

In this work, the MIC of 16 representative antibiotics, encompassing nearly all the pharmacological classes of the so-called critically important antibiotics (CIAs) and highly important antibiotics (HIAs) (WHO, 2017), to 41 *S. thermophilus* isolates derived from raw milk was determined by broth microdilution. Variations in the MIC values in *S. thermophilus* isolates similar to those obtained in the present work have been reported elsewhere for other starter strains (Katla et al., 2001; Temmerman et al., 2003; Tosi et al., 2007). A comparison of the MIC values obtained in the present work and in the literature identified 10 strains thought to harbor antibiotic resistances: seven strains resistant to tetracycline, two resistant to both erythromycin and clindamycin, and one resistant to streptomycin and neomycin. Going beyond the concept of the clinical breakpoint, the idea of the microbiological breakpoint was introduced by Olsson-Liljequist et al. (1997) for the purpose of identifying bacterial strains with acquired and potentially transferable resistance determinants. This term has recently been replaced, as proposed by the European Committee on Antimicrobial Susceptibility Testing [EUCAST (http://www.eucast.org/)], by that of the ecological (or epidemiological) cut-off (the ECOFF). A MIC higher than the ECOFF suggests acquired resistance, which, if due to added genes encoded on mobile genetic elements, have a high risk for horizontal transfer (EFSA, 2012). One of the isolates considered resistant to tetracycline showed a MIC value one dilution higher than the ECOFF (EFSA, 2012). A one dilution deviation of the MIC has been reported to be within the normal intra-laboratory reproducibility range for antibiotic susceptibility assays (Huys et al., 2010). Thus, strains showing MIC values to an antibiotic one dilution above the ECOFF might still represent a susceptible population (EFSA, 2012). A similar conclusion on the number of resistant strains was reached by analysis of the distribution of the MICs. In all but one case, MIC values of antibiotics for which resistant isolates were found followed a bimodal distribution, while in the absence of dedicated resistance mechanisms (intrinsic resistance) statistical normality should be approached (Murray et al., 2003).

Cross-resistance to erythromycin and clindamycin is known as the MLS phenotype (resistance to macrolides, lincosamides, and streptogramines) (Thumu and Halami, 2012) and occurs due to overlapping of the antibiotics' binding sites in the ribosome (Morosini et al., 2012). Streptomycin and neomycin are two aminoglycoside antibiotics with different mode of action. Streptomycin inhibits protein synthesis by binding to the 16S rRNA and thus interfering with the binding of formyl-methionyl-tRNA to the 30S subunit, while neomycin binds to duplex RNA or to triplex RNA-DNA structures (Frieri et al., 2017). Post-transcriptional methylation of 16S rRNA at certain positions can lead to the concomitant resistance to several aminoglycosides, including streptomycin and neomycin (Doi et al., 2016).

Conventional PCR amplification identified *tet*(S) and *ermB* in all strains considered resistant to tetracycline and erythromycin/clindamycin, respectively. *ermB* is the only erythromycin/clindamycin resistance gene that has been detected in *S. thermophilus* so far (Wang et al., 2006; Nawaz et al., 2011). In contrast, several genes for tetracycline resistance have been identified in this species, including genes coding for ribosomal protection proteins, such as *tet*(S) and *tet*(M) (Wang et al., 2006; Ge et al., 2007; van Hoek et al., 2008; Rizzotti et al., 2009), or efflux pumps, such as *tet*(L), and *tet*(A) (Rizzotti et al., 2009; Arioli et al., 2014). Although conventional techniques such as PCR and hybridisation are useful for the characterisation of antibiotic resistance genes and their associated elements, the use of whole genome sequencing and analysis, which is becoming affordable for most laboratories, is rapidly spreading due to its quick response and powerful analytical capabilities (Tait-Kamradt et al., 2009; Flórez et al., 2016; Li et al., 2017). However, due to the short reads obtained with the sequencing technique used (Illumina), and the limitations of current assemblage software, DNA segments containing repeated sequences, such as those of IS-elements (which are abundant close to the antibiotic resistance genes), cannot always be resolved. In the present study, whole genome analysis of the examined five strains confirmed the presence of dedicated antibiotic resistance genes in the tetracycline and erythromycin/clindamycin-resistant strains, but ruled out the presence of such dedicated, added genes responsible for streptomycin/neomycin resistance, as no gene responsible for aminoglycoside resistance of those reported in the literature in LAB species (Ammor et al., 2008; Fraqueza, 2015; Jaimee and Halami, 2016) was seen.

The nucleotide sequence of the St-5 contig carrying *ermB* showed extensive homology to plasmid-derived sequences, particularly to long segments of pRE25 (Schwarz et al., 2001) and pSM19035 (Soberón et al., 2011). pRE25 is a conjugative plasmid that has proven transferable to plasmid-free *E. faecalis* and *Listeria innocua* (Schwarz et al., 2001). It has also been transferred to *Lactococcus lactis* subsp. *cremoris*; however, the plasmid in the transformants was found integrated into the chromosome, suggesting it does not replicate in this species (Schwarz et al., 2001). The involvement of pRE25 in the transfer of *ermB* to *Lactobacillus johnsonii* has already been previously suggested (Flórez et al., 2006). It might be speculated that the presence of antibiotic resistance genes in *S. thermophilus* is due to the promiscuous transfer systems of conjugative plasmids such as pRE25 or pSM19035 (Rizzotti et al., 2009), but the contribution to adaptation (and evolution) of this species through an otherwise inefficient natural competence system (Gardan et al., 2009) cannot be ruled out. In either case, the presence in *S. thermophilus* of clustered regularly interspaced short palindromic repeats (CRISPR) and genes coding for CRISPR-associated systems (CRISPR-Cas systems) (see Supplementary Table 3), which have been shown to degrade phage and plasmid DNA (Garneau et al., 2010), does not seem to be an impenetrable barrier for the incorporation of plasmids segments harboring antibiotic resistance genes.

The whole genetic organization of the contig carrying *ermB* resembled that of ICEs identified in *Streptococcus* species (Beres and Musser, 2007; Camilli et al., 2008; Brenciani et al., 2011; León-Sampedro et al., 2016). ICEs, some of which carry *ermB* genes, are composed of functional modules that correspond to DNA segments and genes or group of genes involved in maintenance and dissemination processes (Carraro and Burrus, 2014). The essential modules of the basic ICE structure are those of integration/excision, replication/DNA processing, DNA secretion, and regulation. Two closely related ICEs -ICE*St1* and ICE*St3*- have already been identified in *S. thermophilus* (Carraro et al., 2011). ICE*St1* was first described as a 35 kbp polymorphic region in the genome of *S. thermophilus* CNRZ 368 (Roussel et al., 1997), while ICE*St3* was initially identified in *S. thermophilus* CNRZ 385 (Pavlovic et al., 2004). These two related elements are also related to other ICEs from different *Streptococcus* species (Carraro and Burrus, 2014). If an ICE is present in St-5, its sequence and structure is completely different to those already reported in *S. thermophilus*. However, whether the ICE is complete or contains additional modules or structures in other contigs remains to be determined.

The greatest risk of the presence in food of LAB harboring transmissible antibiotic resistance genes is the possibility of the latter being transferred to pathogens during food manufacture or during transit through the gastrointestinal tract (Marshall and Levy, 2011). Under the conditions of the present work, neither *tet*(S) nor *ermB* was transferred to *L. delbrueckii*. Since the contig from St-5 carrying *ermB* seemed to harbor all the components of the putative minimal conjugative unit of pRE25 (Schwarz et al., 2001), and this plasmid has been proven transferable in its replicative (as a plasmid) and integrative forms (Schwarz et al., 2001), the transfer of *ermB* from this strain to other species and strains was considered feasible. In the absence of experimental proof to the contrary, the transfer of resistance to other food-borne microorganisms might still be possible, although *S. thermophilus* has previously been shown negative for the transfer of tetracycline resistance to *E. faecalis* and *L. innocua* under similar conjugation conditions (Rizzotti et al., 2009).

## Conclusions

Acquired resistance to tetracycline, erythromycin/clindamycin and streptomycin/neomycin antibiotics was detected in a few strains among 41 *S. thermophilus* isolates derived from raw milk. Correlations were seen between tetracycline and erythromycin/clindamycin resistance and the presence of *tet*(S) and *ermB*, respectively. Genome analysis confirmed the presence of both *tet*(S) and *ermB* and identified IS-related sequences flanking the resistance genes. Somehow, these sequences are thought to be involved in the spread of antibiotic resistance genes in bacteria. Analysis of the contig harboring *ermB* identified a genetic structure very similar to segments of pRE25 from *E. faecalis* and other conjugative plasmids resembling the ICEs in *Streptococcus* species. Although no direct proof was obtained, genome analysis suggested that a deficiency in the methylation of the 16S rRNA molecule, caused by amino acid substitutions in the RsmG methyltransferase, may account for moderate resistance to streptomycin and strong resistance to neomycin in one strain. No transfer of *tet*(S) or *ermB* from *S. thermophilus* to *L. delbrueckii* was observed under yogurt manufacturing and storage conditions. The low phylogenetic relationship of the strains of this study with currently-in-use *S. thermophilus* starters suggests that strains from the same ecosystem free of antibiotic resistances would be a good source for the search of novel starter candidates or starters with novel or improved properties to be used in dairy fermentations.

## Author contributions

BM and ABF: conceived and designed the experiments, performed the experiments and analyzed the data, and wrote and reviewed the manuscript. BM contributed to reagents and materials.

### Conflict of interest statement

The authors declare that the research was conducted in the absence of any commercial or financial relationships that could be construed as a potential conflict of interest. The reviewer MH and handling Editor declared their shared affiliation.
