# Supplementary material for: Antibiotic Resistance-Susceptibility Profiles of Streptococcus thermophilus Isolated from Raw Milk and Genome Analysis of the Genetic Basis of Acquired Resistances
Source: Front Microbiol. 2017 Dec 22;8:2608. doi: 10.3389/fmicb.2017.02608 (PMC5744436; doi:10.3389/fmicb.2017.02608)
Supplement: Supplementary file 1 [file Table1.DOCX]

**Supplementary Table 1.-** General data of the genome sequencing projects of *S. thermophilus* strains resistant to antibiotics isolated from raw milk.

| **Strain** | **Antibiotic resistance, genotype** | **Assembly condition** | **Nº contigs** | **Total bases** | **Contig Max** | **N50** | **N90** |
| --- | --- | --- | --- | --- | --- | --- | --- |
|  |  |  |  |  |  |  |  |
| St-2 | Tetracycline resistant, *tet*(S) | Spades (t99) | 65 | 1,886,531 | 174,559 | 65,326 | 14,340 |
| St-5 | Erythromycin/cindamycin resistant, *ermB* | Spades (t97) | 65 | 1,918,795 | 211,293 | 53,806 | 17,393 |
| St-6 | Erythromycin/clindamycin resistant, *ermB* | Spades (no trimming) | 67 | 1,924,983 | 165,263 | 56,225 | 19,469 |
| St-9 | Tetracycline resistant, *tet*(S) | Spades (t95) | 79 | 1,908,640 | 175,185 | 45,994 | 13,172 |
| St-10 | Streptomycin/neomycin resistant | Spades (no trimming) | 56 | 1,848,642 | 214,067 | 73,583 | 17,896 |
|  |  |  |  |  |  |  |  |
