# Supplementary material for: Antibiotic Resistance-Susceptibility Profiles of Streptococcus thermophilus Isolated from Raw Milk and Genome Analysis of the Genetic Basis of Acquired Resistances
Source: Front Microbiol. 2017 Dec 22;8:2608. doi: 10.3389/fmicb.2017.02608 (PMC5744436; doi:10.3389/fmicb.2017.02608)
Supplement: Supplementary file 2 [file Table2.DOCX]

**Supplementary Table 2.-** General features of the genomes of five antibiotic resistant *S. thermophilus* strains sequenced in this work.

| **Feature/gene(s) coding for** | **Strain** | | | | |
| --- | --- | --- | --- | --- | --- |
|  | **St-2** | **St-5** | **St-6** | **St-9** | **St-10** |
|  |  |  |  |  |  |
| Size (bp) | 1,886,531 | 1,918,795 | 1,924,983 | 1,908,640 | 1,848,642 |
| GC content | 38.7 | 38.7 | 38.7 | 38.7 | 39.0 |
| No. of coding sequences | 2,071 | 2,106 | 2,124 | 2,078 | 2,026 |
| No. subsystems in RAST | 317 | 317 | 317 | 317 | 314 |
| No. of unique genes | 62^a^ | 18 | 19 | 81 | 58 |
| Resistance to antibiotic and toxic compounds | 21^b^ | 15 | 15 | 19 | 15 |
| Antibiotic resistance | *tet*(S) | *ermB* | *ermB* | *tet*(S) | nd^c^ |
| rRNAs | 16S+5S+23S | 16S+5S+23S | 16S+5S+23S | 16S+5S+23S | 16S+5S+23S |
| tRNAs | 39 | 41 | 47 | 41 | 44 |
| Transposases | 9 | 9 | 7 | 9 | 9 |
| Phage-related proteins | 6 | 8 | 6 | 7 | 28 |
| Competence-associated proteins | 24 | 27 | 26 | 26 | 30 |
| CRISPR-associated proteins | 9 (5+4)^d^ | 4 (4) | 4 (4) | 18 (10+4+4) | 18 (10+4+4) |
| Bacteriocins-like substances | 2xBlpU, colicin V, mundticin KS | 2xBlpU, colicin V, mundticin KS, lanthibiotic | 2xBlpU, colicin V, mundticin KS, lanthibiotic | 2xBlpU, colicin V, mundticin KS | 2xBlpU, colicin V, mundticin KS, lanthibiotic |
| Immunity proteins | 3 | 4 | 3 | 3 | 5 |
| 6-phospho-β-glucosidases | 5 (4+1) | 5 (4+1) | 5 (4+1) | 4 (3+1) | 4 (2+2) |
| β-galactosidases | 1 | 1 | 1 | 1 | 1 |
| PrtS-like proteinase | nd | nd | nd | nd | nd |
| Pullulanases | 8 (5+3) | 9 (5+4) | 9 (5+4) | 11 (6+5) | 8 (5+3) |
| Urease activity | 10 (10) | 10 (10) | 10 (10) | 10 (10) | 10 (10) |
| Toxins | Exfoliative toxin, protein J, Doc toxin | Exfoliative toxin, Doc toxin, 2xZeta toxin | Exfoliative toxin, Doc toxin, 2xZeta toxin | Exfoliative toxin, protein J, Doc toxin | Exfoliative toxin |
|  |  |  |  |  |  |

^a^Genes not found in any other *S. thermophilus* sequenced strain among the 37 analyzed (Supplementary Table 1).

^b^RAST category “Virulence, Disease and Defense”, subcategory “Resistance to Antibiotic and Toxic Compounds”.

^c^nd, not detected (absence of recognizable genes)

^d^In parenthesis, number of genes present at different clusters.
