# Supplementary material for: Antibiotic Resistance-Susceptibility Profiles of Streptococcus thermophilus Isolated from Raw Milk and Genome Analysis of the Genetic Basis of Acquired Resistances
Source: Front Microbiol. 2017 Dec 22;8:2608. doi: 10.3389/fmicb.2017.02608 (PMC5744436; doi:10.3389/fmicb.2017.02608)
Supplement: Supplementary file 3 [file Table3.DOCX]

| **Strain** | **Bioproject** | **Assembly** | **Assembly level** |
| --- | --- | --- | --- |
|  |  |  |  |
| *S. thermophilus* JIM 8232 | PRJEA68521 | GCA_000253395.1 | Complete |
| *S. thermophilus* LMG 18311 | PRJNA13162 | GCA_000011825.1 | Complete |
| *S. thermophilus* CNRZ1066 | PRJNA13163 | GCA_000011845.1 | Complete |
| *S. thermophilus* LMD-9 | PRJNA13773 | GCA_000014485.1 | Complete |
| *S. thermophilus* ND03 | PRJNA49149 | GCA_000182875.1 | Complete |
| *S. thermophilus* MN-ZLW-002 | PRJNA159887 | GCA_000262675.1 | Complete |
| *S. thermophilus* ASCC 1275 | PRJNA222865 | GCA_000698885.1 | Complete |
| *S. thermophilus* SMQ-301 | PRJNA251645 | GCA_000971665.1 | Complete |
| *S. thermophilus* MN-BM-A02 | PRJNA274301 | GCA_001008015.1 | Complete |
| *S. thermophilus* MN-BM-A01 | PRJNA293401 | GCA_001280285.1 | Complete |
| *S. thermophilus* S9 | PRJNA307645 | GCA_001514435.1 | Complete |
| *S. thermophilus* KLDS SM | PRJNA325330 | GCA_001663795.1 | Complete |
| *S. thermophilus* CS8 | PRJNA326481 | GCA_001685375.1 | Complete |
| *S. thermophilus* KLDS 3.1003 | PRJNA335873 | GCA_001705585.1 | Complete |
| *S. thermophilus* ND07 | PRJNA327772 | GCA_001855705.1 | Complete |
| *S. thermophilus* APC151 | PRJNA376088 | GCA_002012365.1 | Complete |
| *S. thermophilus* ACA-DC 2 | PRJEB14916 | GCA_900094135.1 | Complete |
| *S. thermophilus* M17PTZA496 | PRJNA230629 | GCA_000521265.1 | Chromosome |
| *S. thermophilus* TH1435 | PRJNA225801 | GCA_000521285.1 | Chromosome |
| *S. thermophilus* TH1436 | PRJNA227503 | GCA_000521305.1 | Chromosome |
| *S. thermophilus* MTH17CL396 | PRJNA230626 | GCA_000521325.1 | Chromosome |
| *S. thermophilus* TH982 | PRJNA231305 | GCA_000572065.1 | Chromosome |
| *S. thermophilus* TH1477 | PRJNA231320 | GCA_000572095.1 | Chromosome |
| *S. thermophilus* 1F8CT | PRJNA231319 | GCA_000836595.1 | Chromosome |
| *S. thermophilus* TH985 | PRJNA231300 | GCA_000836675.1 | Chromosome |
| *S. thermophilus* KLDS3.1012 | PRJNA291967 | GCA_001266475.1 | Scaffolds |
| *S. thermophilus* MTCC 5461 | PRJNA71269 | GCA_000335495.1 | Contig |
| *S. thermophilus* MTCC 5460 | PRJNA71267 | GCA_000335515.1 | Contig |
| *S. thermophilus* C106 | PRJNA288538 | GCA_001306395.1 | Contig |
| *S. thermophilus* UC8547del3 | PRJNA335822 | GCA_001867185.1 | Contig |
| *S. thermophilus* UC8547 | PRJNA335822 | GCA_001867245.1 | Contig |
| *S. thermophilus* CNCM I-1630 | PRJNA67867 | GCA_000284675.1 | Contig |
| *S. thermophilus* St-2 |  |  | Contig |
| *S. thermophilus* St-5 |  |  | Contig |
| *S. thermophilus* St-6 |  |  | Contig |
| *S. thermophilus* St-9 |  |  | Contig |
| *S. thermophilus* St-10 |  |  | Contig |
|  |  |  |  |

**Supplementary Table 3.-** Genome sequences of the *Streptococcus thermophilus* strains used in the phylogenetic analysis.

Available *S. thermophilus* genome sequences were retrieved from GenBank (<https://www.ncbi.nlm.nih.gov/genome/genomes/420>; assessed in August 2017).
