# Supplementary material for: Antibiotic Resistance-Susceptibility Profiles of Streptococcus thermophilus Isolated from Raw Milk and Genome Analysis of the Genetic Basis of Acquired Resistances
Source: Front Microbiol. 2017 Dec 22;8:2608. doi: 10.3389/fmicb.2017.02608 (PMC5744436; doi:10.3389/fmicb.2017.02608)
Supplement: Supplementary file 4 [file Table4.DOCX]

**Supplementary Table 4**.- Analysis of the open reading frames (ORFs) and other features identified in the contigs from the genome sequence of *S. thermophilus* St-5 (28,150 bp) and *S. thermophilus* St-9 (3,784 b9) harbouring the erythromycin/clindamycin and tetracycline resistance genes *ermB* and *tet*(S), respectively.

| **ORF/feature** | **5’ end position** | **3’ end position^a^** | **% GC content** | **No. of aa^b^** | **Known protein with the highest homology (microorganism)^c,d^/structure** | **% aa identity (identity length/total length)** | **GenBank Accession no.** |
| --- | --- | --- | --- | --- | --- | --- | --- |
|  |  |  |  |  |  |  |  |
| ***S. thermophilus* St-5 (erythromycin/clindamycin resistant strain)** | | | | | | | |
| ΔORF1 | 52 | 372 | 39.8 | 106 | IS6 family transposase (*Enterococcus faecium*) | 100% (106/106) | WP_060797527.1 |
| ORF2 | 668 | 752 | 29.8 | 27 | **MLS leader peptide (MLVFQMRNVDKTSTVLKQTKNSDYADK)** | 100% (27/27) | WP_001038795.1 |
| ORF3 | 877 | 1614 | 33.1 | 245 | **23S rRNA [adenine(2058)-N(6)]-methyltransferase, ErmB (Bacteriaa)** | 100% (245/245) | WP_001038795.1 |
| ORF4 | 1760 | 2044 | 32.6 | 94 | Truncated topoisomerase (Bacteria) | 100%(94/94) | WP_076605238.1 |
| Inverted repeat (IR) | 2095 | 2141 | - | - | 20 bp-N7-20 bp | - | - |
| ORF5 | 2,147 | 3,043 | 31.7 | 298 | Partitioning delta (δ) protein, ParA from pRE35 (*Enterococcus faecalis*) | 100% (298/298) | WP_002326827.1 |
| IR | 3,044 | 3,092 | - | - | 21 bp-N7-21 bp | - | - |
| ORF6 | 3,135 | 3,350 | 33.8 | 71 | Peptide-binding omega (ω) protein (Firmicutes) | 100% (71/71) | WP_001835296.1 |
| ORF7 | 3,368 | 3,640 | 29.6 | 90 | Epsilon (ϵ) antitoxin (Bacilli) | 100% (90/90) | WP_002326825.1 |
| ORF8 | 3,642 | 4,505 | 37.5 | 287 | Toxin zeta (ζ) (Firmicutes) | 100% (287/287) | WP_002332783.1 |
| IR | 4,518 | 4,555 | - | - | 15 bp-N7-15 bp | - | - |
| ORF9 | 4,897 | 5,262 | 30.6 | 121 | Hypothetical protein (*E. faecium*) | 99% (120/121) | ERK34973.1 |
| ORF10 | 5,787 | 6,293 | 31.8 | 168 | Hypothetical chaperon protein, DnaJ (Lactobacillales) | 100% (168/168) | WP_011666354.1 |
| ORF11 | 6,327 | 6,560 | 26.0 | 77 | Thymidylate synthase, ThyX (*E. faecalis*) | 100% (77/77) | OOP50726.1 |
| IR | 6,631 | 6,680 | - | - | 23 bp-N3-23 bp | - | - |
| ORF12 | 6,911 | 6,654 | 26.0 | 85 | Hypothetical protein (Bacilli) | 100% (72/72) | WP_000002668.1 |
| ORF13 | 7,213 | 6,914 | 23.0 | 99 | Hypothetical protein (Lactobacillales) | 100% (99/99) | WP_002332653.1 |
| *oriT* | 7,369 | 7,407 | 44.8 | - | *oriT* sequence from pRE25 (*E. faecalis*) (AGGGCGCACTTATACGCAGTAACTTCGTTACTTCGTAT) | 100 (38/38) | NC_008445.1 |
| ORF14 | 7,496 | 9,490 | 28.8 | 664 | Mobilization protein, site-specific, single-stranded-nuclease, MobA (*E. faecalis*) | 99% (660/661) | WP_033626033.1 |
| ORF15 | 9,514 | 9,846 | 39.9 | 110 | Hypothetical protein, TrsB (Bacilli) | 100% (110/110) | WP_000713503.1 |
| ORF16 | 9,865 | 10,248 | 35.7 | 127 | Hypothetical protein, TrsC (Bacilli) | 100% (127/127) | WP_002332651.1 |
| ORF17 | 10,265 | 10,894 | 33.5 | 209 | Hypothetical protein, TrsD (*Enterococcus* spp.) | 99% (209/209) | WP_002332650.1 |
| ORF18 | 10,905 | 12,866 | 32.7 | 653 | Hypothetical ATPase, protein TrsE (*E. faecium*) | 100% (653/653) | WP_002332649.1 |
| ORF19 | 12,880 | 14,232 | 30.2 | 450 | Conjugal transfer protein, TrsF (*Enterococcus gallinarum*) | 100% (450/450) | WP_002332648.1 |
| ORF20 | 14,254 | 15,363 | 38.9 | 369 | Putative lytic transglycosidase (amidase) (*E. faecium*) | 100% (369/369) | WP_002332647.1 |
| ORF21 | 15,376 | 15,927 | 31.7 | 183 | Hypothetical protein (*Enterococcus*) | 100% (183/183) | WP_002332646.1 |
| ORF22 | 15,932 | 16,363 | 32.7 | 144 | Hypothetical protein (*Enterococcus*) | 100% (144/144) | WP_002332645.1 |
| ORF23 | 16,356 | 18,011 | 34.8 | 551 | TraG-/VirD4-like, type IV secretory protein, TrsK (*E. faecalis*) | 100% (551/551) | CAD44390.1 |
| ORF24 | 18,029 | 18,952 | 37.1 | 307 | Hypothetical conjutative protein (*E. faecalis*) | 100% (307/307) | CAD44391.1 |
| ORF25 | 18,952 | 19,884 | 38.5 | 310 | Hypothetical protein, TrsL (*E. faecalis*) | 100% (310/310) | CAD44392.1 |
| ORF26 | 19,901 | 20,869 | 31.4 | 322 | Hypothetical eta (η) protein, (*E. faecalis*) | 100% (322/322) | CAD44393.1 |
| ORF27 | 20,898 | 21,266 | 29.0 | 122 | Hypothetical theta (θ) protein (Bacilli) | 100% (122/122) | WP_000516429.1 |
| ORF28 | 21,326 | 22,147 | 45.3 | 273 | Hypothetical tau (τ) protein (*Carnobacterium gallinarum*) | 100% (273/273) | WP_034558104.1- |
| Direct repeats (DRs); iterons | 21,478 | 21,848 | - | - | DR1 (TCCAGTTGA) x 8; one base (T); DR2 (CCAACAGAG) x 12; DR3 (CCAACGGAA) x 21; DR2 and DR3 interspersed. | - | - |
| ORF29 | 22,427 | 22,705 | 33.3 | 92 | Plasmid copy number control, CopS (Firmicutes) | 100% (92/92) | WP_024407057.1 |
| IR | 22,687 | 22,727 | - | - | 16 bp-N9-16 bp | - | - |
| ORF30 | 23,086 | 24,576 | 35.5 | 496 | Replication protein RepS from pSM19035 (*Streptococcus agalactiae*) | 100% (496/496) | WP_010890218.1 |
| ORF31 | 24,923 | 25,093 | 29.8 | 56 | Hypothetical protein, Orfα (Lactobacillales) | 100% (56/56) | WP_000713595.1 |
| ORF32 | 25,107 | 25,724 | 35.2 | 205 | Hypothetical protein, Orfβ (site-specific recombinase) (Bacilli) | 100% (205/205) | WP_001062587.1 |
| ORF33 | 25,724 | 27,868 | 35.9 | 714 | Hypothetical protein, Orfγ (topoisomerase I) (Bacilli) | 100% (714/714) | WP_000108743.1 |
| DR | 27,902 | 27,944 | - | - | 14 bp imperfect DR (TTATCACAAATCAC) x 3 | - | - |
| IR | 27,933 | 27,948 | - | - | 12 bp-N2-12 bp | - | - |
|  |  |  |  |  |  |  |  |
| ***S. thermophilus* St-9 (tetracycline resistant strain)** | | | | | | | |
| ΔORF1 | 1 | 105 | 31.4 | 35 | IS1191 transposase (IS256 family) (*Streptococcus*) | 97% (34/384) | ERJ79159.1 |
| IR | 920 | 957 | - | - | 16 bp-N7-16 bp | - | - |
| ΔORF2 | 922 | 978 | 20.5 | 18 | **Tetracycline resistance determinant leader peptide (NPSDKSIYHWDFFCFLSF)** | 60% (15/38) | WP_001791010.1 |
| *tet*(S) | 979 | 2,916 | 33.3 | 646 | **Tetracycline resistant protein Tet(S) (Bacteria)** | 100% (646/646) | AIB06868.1 |
| ORF4 | 2,948 | 3,125 | 30.5 | 55 | Conjugal transfer protein (Bacilli) | 98% (54/55) | WP_005228365.1 |
| ΔORF5 | 3,444 | 3,783 | 37.5 | 113 | IS6 family transposase (*Enterococcus*) | 99% (112/262) | OTO31657.1 |
|  |  |  |  |  |  |  |  |

^a^Including start and stop codons.

^b^aa, amino acids.

^c^Colour code of ORFs: in dark blue, ORFs involved in antibiotic resistance; in yellow, complete and/or incomplete ORFs related to transposases, invertases, or topoisomerases; in light blue, ORFs related to plasmid segregation and stability; in green, module components for plasmid mobilization; in red, ORFs involved in plasmid replication and control; in white, ORFs encoding hypothetical proteins, other proteins, and direct and inverted repeats.

^d^Identity of the proteins followed the designation for those equivalent in pRE25 (Schwarz et al., 2001) and pSM19035 (Volante et al., 2014).
