# Supplementary material for: Antibiotic Resistance-Susceptibility Profiles of Streptococcus thermophilus Isolated from Raw Milk and Genome Analysis of the Genetic Basis of Acquired Resistances
Source: Front Microbiol. 2017 Dec 22;8:2608. doi: 10.3389/fmicb.2017.02608 (PMC5744436; doi:10.3389/fmicb.2017.02608)
Supplement: Supplementary file 5 [file DataSheet1.PDF]

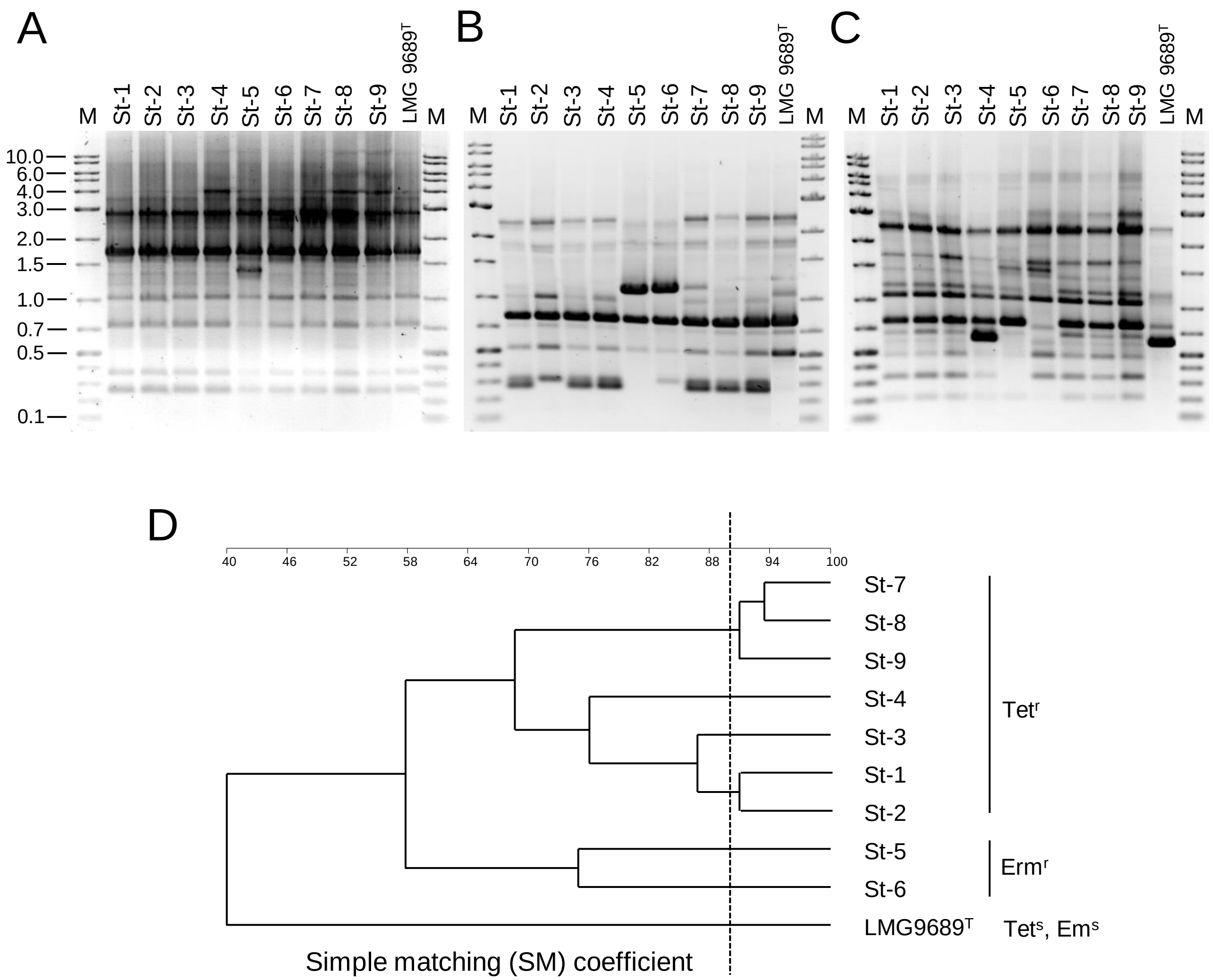

Supplementary Figure 1.- Different rep-PCR and RAPD typing profiles, obtained with primers BoxA2R (Panel A), M13 (Panel B) and OPA18 (Panel C), for wild strains of *Streptococcus thermophilus* isolates from milk and traditional cheeses. Below the panels, dendogram of similarity of the combined typing profiles expressed by the Simple Matching (SM) coefficient. Clustering was performed by the unweighted pair group method using arithmetic averages (UPGMA). In bold, strains subjected to genome sequencing.
