# Supplementary material for: Antibiotic Resistance-Susceptibility Profiles of Streptococcus thermophilus Isolated from Raw Milk and Genome Analysis of the Genetic Basis of Acquired Resistances
Source: Front Microbiol. 2017 Dec 22;8:2608. doi: 10.3389/fmicb.2017.02608 (PMC5744436; doi:10.3389/fmicb.2017.02608)
Supplement: Supplementary file 6 [file DataSheet2.PDF]

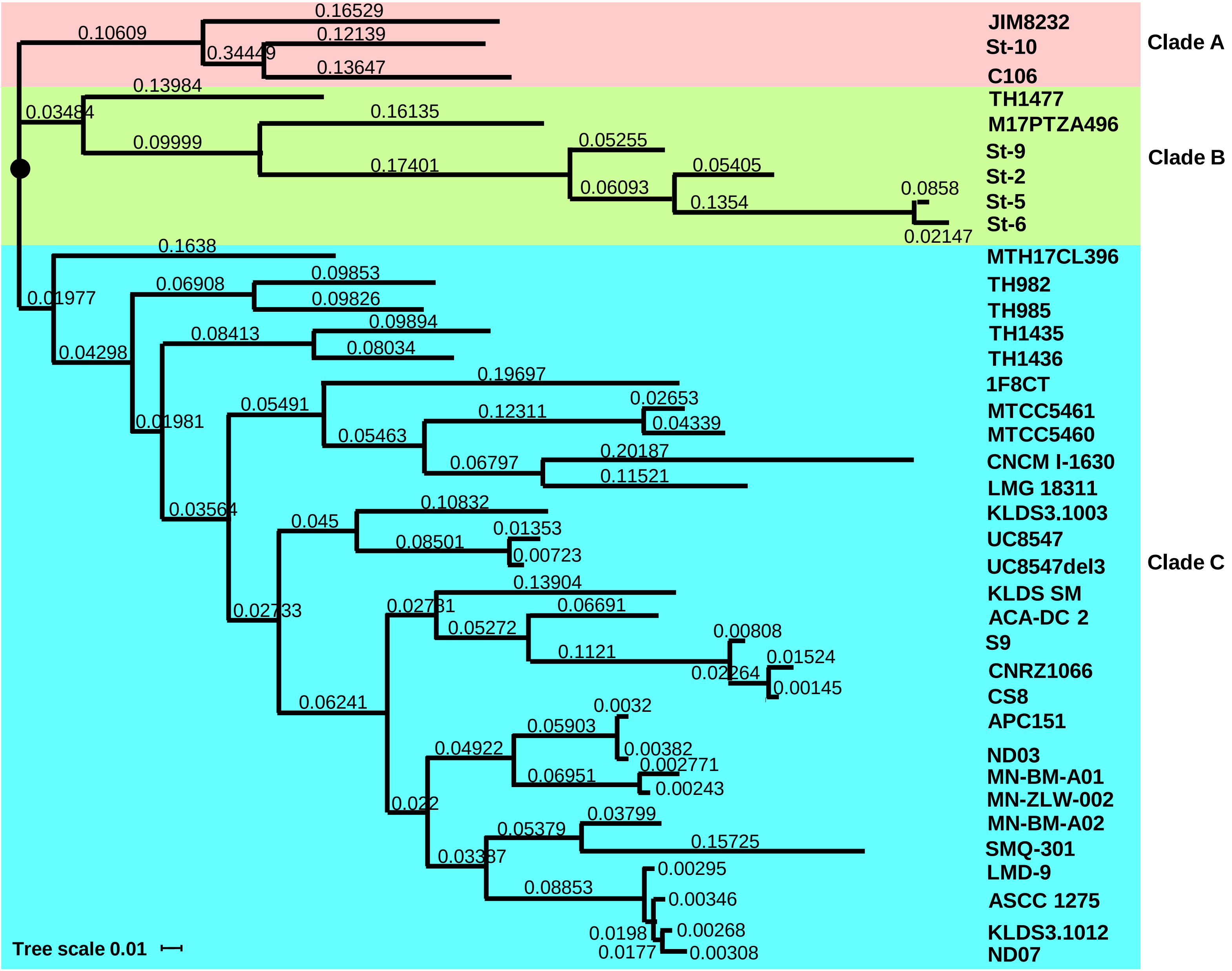

Supplementary Figure 2.- Phylogenomic analysis of 32 representative strains of *Streptococcus thermophilus* available at the NCBI database (https://www.ncbi.nlm.nih.gov/genome/genomes/420; assessed in August 2017) and the five wild *S. thermophilus* antibiotic resistant strains analysed in this study, based on the gene content obtained with Roary software and visualized using iTOL.
