# Supplementary material for: Antibiotic Resistance-Susceptibility Profiles of Streptococcus thermophilus Isolated from Raw Milk and Genome Analysis of the Genetic Basis of Acquired Resistances
Source: Front Microbiol. 2017 Dec 22;8:2608. doi: 10.3389/fmicb.2017.02608 (PMC5744436; doi:10.3389/fmicb.2017.02608)
Supplement: Supplementary file 7 [file DataSheet3.PDF]

A

Yoghurt batch	Yoghurt starters (mating strains)		Antibiotic resistance gene in *S. thermophilus*	Transference
Y1	*S. thermophilus* St-1			-
Y2	*S. thermophilus* St-3		*tet*(S)	-
Y3	*S. thermophilus* St-9	*L. delbrueckii* subsp. *bulgaricus* CECT 4005		-
Y4	*S. thermophilus* St-5		*ermB*	-
Y5	*S. thermophilus* St-6			-
Y6	*S. thermophilus* St-1			-
Y7	*S. thermophilus* St-3		*tet*(S)	-
Y8	*S. thermophilus* St-9	*L. delbrueckii* subsp. *bulgaricus* LMG 6901		-
Y9	*S. thermophilus* St-5		*ermB*	-
Y10	*S. thermophilus* St-6			-

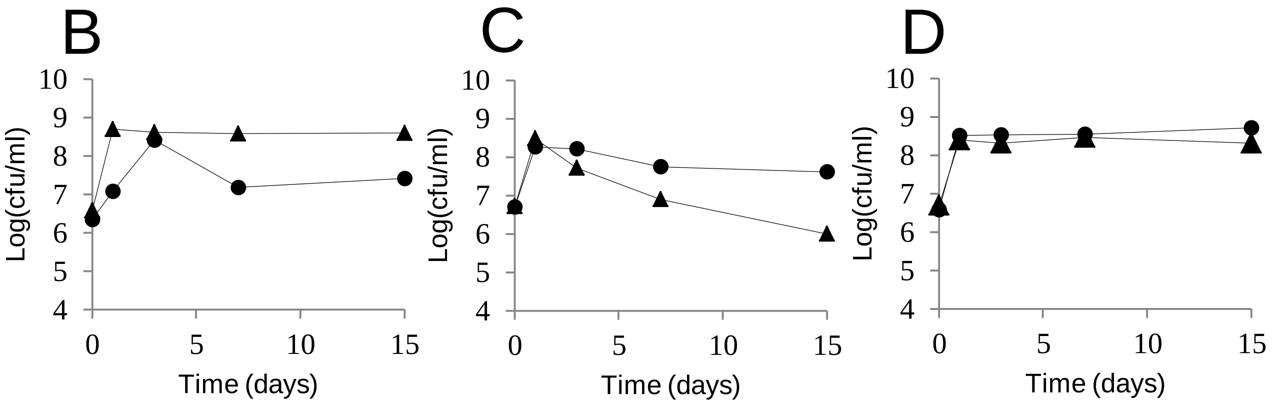

Supplementary Figure 3.- Experimental batches of yoghurts indicating the starter strains and the antibiotic resistance genes (Table A). Microbiological evolution of *S. thermophilus* (black triangles) and *L. delbrueckii* (black circles) in individual batches, such as Y1 (Panel B) and Y8 (Panel C), and average results for Y2, Y3, Y4, Y5, Y6, Y7, Y9 and Y10 (Panel D).
